# Current Landscape: The Mechanism and Therapeutic Impact of Obesity for Breast Cancer

**DOI:** 10.3389/fonc.2021.704893

**Published:** 2021-07-19

**Authors:** Chongru Zhao, Weijie Hu, Yi Xu, Dawei Wang, Yichen Wang, Wenchang Lv, Mingchen Xiong, Yi Yi, Haiping Wang, Qi Zhang, Yiping Wu

**Affiliations:** Department of Plastic Surgery, Tongji Hospital, Tongji Medical College, Huazhong University of Science and Technology, Wuhan, China

**Keywords:** breast cancer, obesity, adipokines, risk, outcomes, therapy

## Abstract

Obesity is defined as a chronic disease induced by an imbalance of energy homeostasis. Obesity is a widespread health problem with increasing prevalence worldwide. Breast cancer (BC) has already been the most common cancer and one of the leading causes of cancer death in women worldwide. Nowadays, the impact of the rising prevalence of obesity has been recognized as a nonnegligible issue for BC development, outcome, and management. Adipokines, insulin and insulin-like growth factor, sex hormone and the chronic inflammation state play critical roles in the vicious crosstalk between obesity and BC. Furthermore, obesity can affect the efficacy and side effects of multiple therapies such as surgery, radiotherapy, chemotherapy, endocrine therapy, immunotherapy and weight management of BC. In this review, we focus on the current landscape of the mechanisms of obesity in fueling BC and the impact of obesity on diverse therapeutic interventions. An in-depth exploration of the underlying mechanisms linking obesity and BC will improve the efficiency of the existing treatments and even provide novel treatment strategies for BC treatment.

## Introduction

Breast cancer (BC) is ranked as the most common malignancy and the major leading cause of cancer-related death among women worldwide (1). The main reason is that advanced BC is frequently confronted with a high rate of relapse, invasion, and metastasis, especially the organotropic metastasis capacity to the brain and lung (2). Obesity is a pivotal lifestyle driver of the current and prospective BC rates. According to the individually calculated body mass index (BMI), the World Health Organization (WHO) has defined that obesity class I-III has a BMI ≥ 30 kg/m^2^ at baseline and BMI < 30 kg/m^2^ is considered non-obese (3). Numerous epidemiological studies have authenticated that obesity is an important risk factor for BC, which means that obesity is not only associated with the incidence of BC, but also related to poor prognosis, high incidence, and decreased survival rate in BC patients (4).

Obesity is defined as a chronic disease induced by an imbalance of energy homeostasis. In obesity, metabolic disorders occur in adipose tissues, leading to the secretion of many pro-inflammatory cytokines, growth factors, and hormones, which in turn contribute to the formation of the tumor microenvironment (TME) and the progression of cancer within the breast tissue. Although biological explanations of how obesity affects BC have been widely discussed, the role of obesity during BC initiation and development remains incompletely mapped and being constantly updated. Like other types of cancer, until today, the main treatment of BC is still surgical resection and radiotherapy, which is an indispensable part of the treatment of local lesions. The metastasis and occurrence of BC continue to lead to unacceptable cancer-related deaths, leading to the diverse methods of chemotherapy, hormone therapy, and immunotherapy. Existing researches have shown that obesity can affect the effectiveness of conventional BC treatments and further lead to therapeutic resistance. This article will summarize the latest studies on the functions and mechanisms of obesity in BC, including the disorders of adipokines, insulin, and insulin-like growth factor (IGF), endogenous sex hormones, chronic inflammation. It also summarized the impact of obesity on BC therapy, such as surgery, radiotherapy (RT), chemotherapy, endocrine therapy (ET) and immunotherapy, and weight management in BC. An in-depth understanding of the mechanisms linking obesity and BC, as well as the impact of obesity on the effectiveness and tolerance of BC treatment, is paramount for establishing new strategies for the prevention and therapeutic interventions of BC.

## Obesity and the Risk Factors of Breast Cancer

The excess weight gain of women during adulthood can increase the risk of BC, and can even lead to an earlier onset of BC and a worse prognosis (5). Patients hospitalized for overweight and obesity are associated with an increased risk of several specific cancers, including BC, compared to the general population (6). Among obese women, BC risk factors have been found to differ in menopausal status, tumor subtypes, and some other conditions.

The relationship between obesity and BC is intricate. The associations appeared to be extremely consistent for postmenopausal BC that high obesity is a risk factor for BC in postmenopausal women independent of ethnic factors. Park et al. demonstrated that in postmenopausal women in Korea, there was a positive relationship between obesity and BC (7). This may attribute to the higher estrogen levels produced in adipose tissue in postmenopausal women (8). However, there is still controversy on their impact on the risk of premenopausal BC. The obesity impact on premenopausal BC risk might be a positive correlation, negative correlation, or even irrelevant, which is involved in ethnic differences or/and sample size of the clinical trial. The fact that many previous studies performed in the Western population with a negative association and Asian studies showed uncertain associations between obesity and BC, indicates the ethnic and racial importance in premenopausal BC risk. For example, a systematic analysis of premenopausal BC suggested a negative association of obesity and BC risk in premenopausal Caucasian and African women (9). Similarly, the investigation among over 6 million Korean women in 2020 suggested that there was a negative association between obesity and BC in premenopausal women (7). However, multiple studies in Asian premenopausal women indicated no significant effect of obesity on the risk of BC during the premenopausal period (10) (11). In addition, Jeong et al. demonstrated an increased risk of triple-negative BC (TNBC) in obese type II (BMI ≥ 30 kg/m^2^) premenopausal Korean women (12). This study was consistent with the finding of a meta-analysis, which revealed the significant positive association of obesity and overweight with BC during the premenopausal period in Asian women (13).

Epidemiological data suggested that large disparities are presented in the prevalence of obesity and overweight among women in different populations. In general, different ethnic groups vary in body size, body composition, and fat composition, and body fat distribution, which might result in subtle differences in metabolic levels and BC risk. Compared with other ethnic groups, Asians seem to have higher overall fat levels and abdominal fat as well as lower lean body mass for a given BMI (14). Amadou et al. showed that an increase of 5 kg/m^2^ in BMI was associated with a significant 5% reduction in premenopausal BC risk (9). Especially, there was an inverse relationship between obesity and the risk of premenopausal BC only among Africans and Caucasians, but there was a significant positive association was observed among Asian women (9). Briefly, the determinants of obesity are complex and diverse, which is concerned with biological alterations, socio-economic and other environmental and behavioral influences. And, ethnic and racial differences are a nonnegligible key factor in obesity and BC risk.

The effect of obesity on BC also varies with different BC subtypes. In obesity type II (BMI≥30 kg/m^2^), premenopausal women had an increased risk of TNBC, and postmenopausal women had an increased risk of Luminal A, Luminal B, HER2– BC, and TNBC (12). Furthermore, a retrospective investigation of clinicopathological BC features of postmenopausal Japanese women indicated that compared with obese patients, mean values of the Ki67 labeling index were significantly higher in lean patients, and HER2+ tumors were more often found in lean patients (15). More aggressive tumors were observed in lean postmenopausal women, which contradicted the prevailing perception of BC in obese women. In order to ascertain the relationship between obesity and BC risk in more refined tumor subtypes, Nattenmüller et al. evaluated six well-established immunohistochemical markers in BC samples (16). It suggested that obesity was related to the risk of breast tumors with lower aggressiveness such as ER+, PR+, HER2–, Ki67low, Bcl-2+, and p53– tumors in postmenopausal women. Further mechanistic studies are required to underlie the associations, as well as larger-scale analyses of the pooled prospective cohort to investigate relationships between obesity and BC subtypes in more detail.

In addition to the traditional epidemiological studies, Shu et al. conducted Mendelian randomization analyses to assess the connection of BC risk with BMI and other indicators, using genetic instruments (17). They found genetically predicted obesity was associated with the BC risk regardless of age, menopausal status, ER status, and family history, unveiling the complex inter-relations of genetics, obesity, and BC risk. Nevertheless, Being overweight might not sufficient for the development of BC, which may be associated with LEP/LEPR gene polymorphisms. Liu et al. conducted a large case-control study among females in southwestern China and showed that persistent overweight (BMI ≥ 24 kg/m^2^) along with LEPrs7799039 AA or LEPRrs1137100 GG genotypes synergistically increased the risk of BC (18). Bariatric surgery has been deemed as the most effective manner for obese patients to lose weight in both the short and long term, and is more effective in producing sustained weight loss than dieting. It was interesting that bariatric surgery was significantly relevant to reduced BC risk, both for cancer incidence and mortality, by a meta-analysis (19). Finally, further research into the complex association of obesity and BC risk will help to guide BC prevention.

## Obesity and Outcomes of Breast Cancer

As Vernaci et al. proposed that, there was a negative impact of obesity on the prognosis in a population of BC patients (20). Over a long period of follow-up, a high BMI was associated with increased rates of relapse, second primary tumors, and death occurrence. The effect of BMI on the prognosis of BC depends on many factors, including tumor subtype, menopausal status, and age. In HR+ HER2– patients, obesity played a negative prognostic role in both pre-menopausal and post-menopausal women in the Asian population (21). Besides, in a cross-sectional study, compared with women of normal weight, obese and morbidly obese women were affected by advanced HER2+ BC with histological grades 2 or 3 (22). Wang et al. found that in Hebei, China, BC patients with a high BMI were at greater risk of poor prognosis than BC patients with a low BMI, especially in patients over 50 years of age (23). One possible key to improving BC outcomes is to maintain an appropriate BMI for BC patients. However, the conclusion regarding the role of obesity as a prognostic indicator in BC individuals remains inconsistent worldwide. The study of Cacho-Díaz et al. reported that no association was found between overall survival (OS) and either patient with brain metastases from BC (BMBC) with a BMI > 25 kg/m^2^ or normal weight in Mexico (24). A study conducted in New Zealand showed that obesity was not associated with inferior locoregional control or survival outcomes, supporting the practice of continuing to offer breast-conserving treatment for women regardless of BMI (25). Thus, studies are still needed to continue maturing the definition of BMI in predicting BC prognosis under different situations. On all accounts, this will ultimately provide a reliable basis for the individualized prevention and treatment of BC.

## Mechanisms Linking Obesity and Breast Cancer

**Figure 1:**
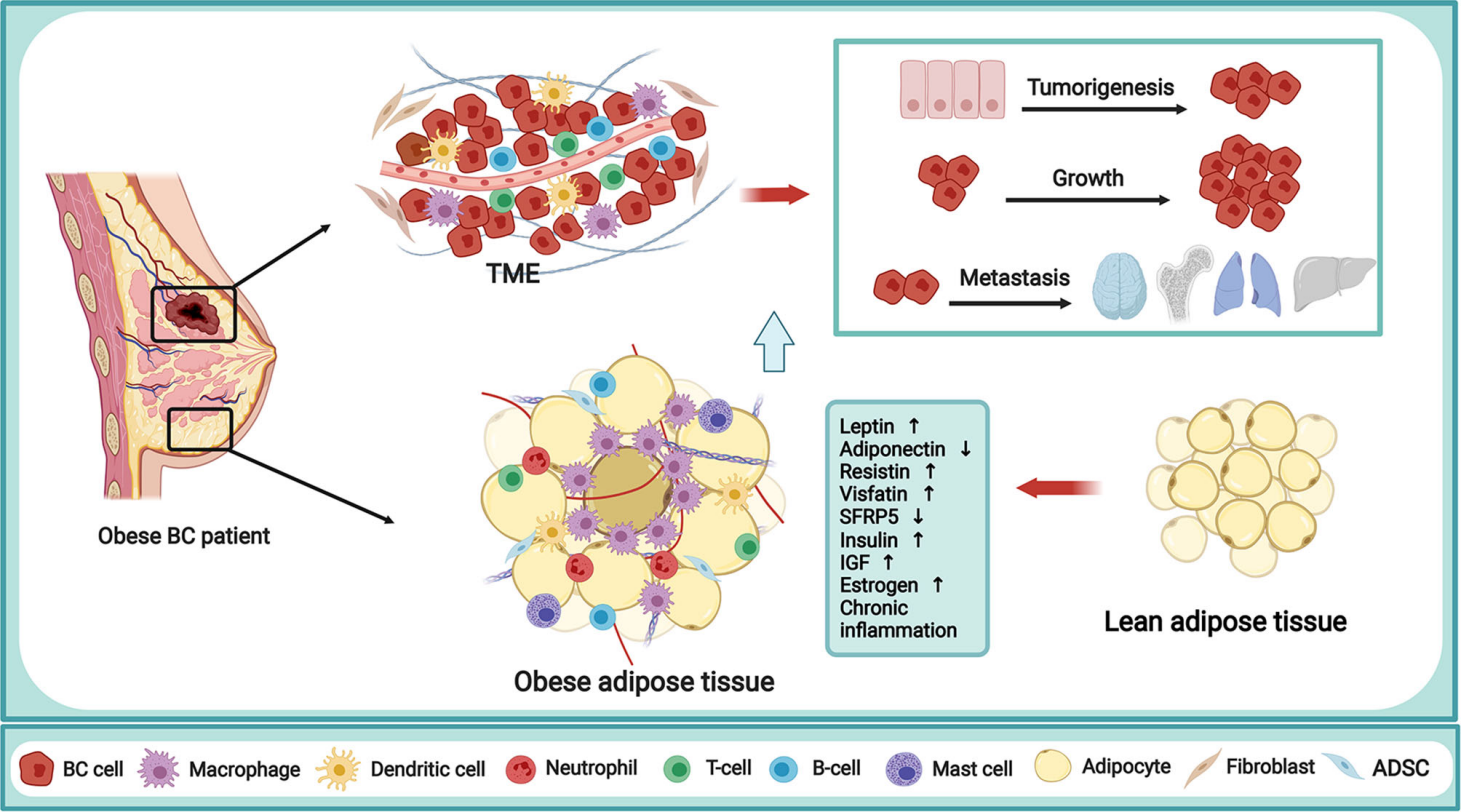
Mechanism of obesity in fueling breast cancer. Obesity is a complex abnormal state, accompanied by various alterations capable of regulating the behavior of BC cells and TME. The obese adipose tissue in BC patient is directly related to the secretion of multiple adipokines, increased levels of insulin, IGF and endogenous sex hormone, and the chronic inflammation state, thus creating a TME that encourages tumorigenesis, growth and metastasis of BC. BC, breast cancer; TME, tumor microenvironment; IGF,insulin-like growth factor; SFRP5, secreted frizzled-related protein 5; ADSC, adipose-derived stem cells. The figure was created with BioRender.com.

It is well-acknowledged that there is an intensive epidemiological link between obesity and BC incidence, but the underlying mechanism is very complicated in the obesity-driven BC progress. The increased or dysfunctional adipose tissue is directly associated with increased levels of many adipose-derived factors, thus creating an environment that encourages BC cancerization. Nowadays, the proposed mechanisms in this process mainly include adipokines, insulin and IGF, sex hormone, and chronic inflammation, in which dysregulation can increase BC incidence, progression, and worsen clinical outcomes (**Figure 1**). It is interesting to note that obesity reinforces the activation of the inflammatory cascade for BC carcinogenesis. Concretely, the adipokines, such as leptin, adiponectin, resistin, and other adipokines like visfatin, secreted frizzled-related protein 5 (SFRP5), play an indispensable role in the manipulation of obesity-associated BC. Overall, these factors contribute to the obesity-induced pro-inflammatory milieu, as well as the crosstalk between adipocytes, immune cells, and breast epithelial cells, which are beneficial for BC risk. The thorough elucidation of the link between obesity and BC will undoubtedly provide the key clues for BC prevention and treatment.

### Adipokines

Adipokines are soluble factors produced by adipocytes, including a large proportion of bioactive metabolites, lipids, and bioactive peptides, and over 600 adipokines have been identified so far. In the obesity state, the levels of adipokines are usually in an imbalanced state. The obesity-induced adipokines play a critical and complex role in driving the malignant phenotype of BC *via* endocrine, paracrine, and autocrine pathways. Among them, leptin and adiponectin are the most studied classic adipokines associated with obesity-triggered BC progression. It was reported that the increased level of leptin in obesity could promote BC initiation, progression, metastasis, and resistance to therapy through a variety of mechanisms including the activation of PI3K/Akt, JAK/AKT/STAT, and FAK-Src signaling pathway. In contrast, obesity results in a decreased expression level of adiponectin, which in turn accelerates the BC risk and progression. Notably, other adipokine disorders caused by obesity, including resistin, visfatin, and SFRP5, are also proved to be key orchestrators in BC oncogenesis and progression. The specific implicated mechanisms of main adipokines in regulating obesity-related BC were listed in **Table 1**.

**Table 1:** The roles of adipokines in linking obesity and BC.

Adipokine	Model	Function	Mechanism	Ref.
Leptin	3D cell coculture model (HMT-3522 S1 mammary epithelial cells); DIO murine model	Leptin could promote BC initiation.	Leptin mediated the loss of apical polarity and promoted premalignant alterations of the mammary gland by activation of PI3K/Akt signals.	(26)
	Mammary epithelial cell models (HMEC, MCF7 and MDA-MB-231)	Leptin could modulate the oxidative status of BC.	Leptin modulated the oxidative status of mammary epithelial cells differently according to their neoplastic state.	(27)
	Cultured cell model of BC (MCF7 and MDA-MB-231)	Leptin could promote BC migration and invasion.	Leptin induced cell migration and invasion in a FAK-Src-dependent manner in BC cells.	(28)
	Cultured cell model of BC (MCF-7, ZR-75-1 and MDA-MB-231)	Leptin could induce BC motility, migration and invasion.	Leptin induced cell viability, EMT, sphere-forming ability, and migration of ERα+ BC cells which was mediated by inhibiting CCN5 signaling *via* activating JAK/AKT/STAT-pathway.	(29)
	Cultured cell model of BC (BT20, MDA-MB-231, MDA-MB-468, MCF7 and HCC1806); TNBC PDX model	Leptin could induce BC migration and metastasis.	Leptin produced by obASCs mediated EMT in *vitro* and promoted tumor metastasis in *vivo*.	(30)
	Cultured cell model of BC (MCF7 and MDA-MB-231)	Leptin could encapsulate in EVs induced BC proliferation, migration and invasion.	leptin encapsulated in EVs derived from obese adipose tissue, thereby mediating pro-tumoral activities and malignancy phenotype of BC cells.	(31)
	Cultured cell model of BC (E-Wnt, M-Wnt and MDA-MB-231); DIO MMTV-Wnt-1 transgenic murine model	Leptin could induce BC viability, migration, invasion, CSC enrichment and EMT.	Increased leptin signaling was causally linked to obesity-associated TNBC development by promoting CSC enrichment and EMT.	(32)
	Co-culture model	Leptin could induce a pro-angiogenic effect on BC.	VEGFA was up-regulated in macrophages after exposure to adipocytes through releasing leptin.	(33)
	Cultured cell model of BC (4T1); DIO murine BC model (4T1); human TNBC patients	Leptin could induce BC progression and metastasis.	The leptin gene expression was negatively correlated with the infiltration of tumor-reactive CD8+ T cells in human TNBC tumors from obese patients when compared to non-obese.	(34)
	Cultured cell model of BC (Py8119); DIO murine BC model (Py8119)	Leptin could increase the oxidation of fatty acids and BC progression.	Leptin-enriched mammary adipocytes and fat tissues downregulated CD8+ T cell effector functions through activating STAT3-FAO and inhibiting glycolysis.	(35)
	DIO murine BC model (4T1)	Leptin could promote BC progression.	Leptin facilitated the MDSCs accumulation, while MDSCs down-regulated the leptin production. HFD-induced MDSCs participated in tumor growth facilitation by inhibiting lethal CD8+ T cells.	(36)
	Cultured cell model of BC (MDA-MB-231, BT-20, MCF7 and MDA-361)	Leptin could promote BC resistance to immune attacks.	Leptin could drive the tumor to escape from immune attacks by enhancing fatty acid oxidation and tumor resistance to NK cell lysis *via* PGC-1 activation.	(37)
	Cultured cell model of BC (MCF-7 and SK-BR-3)	Leptin could promote bone metastasis of BC.	Leptin promoted bone metastasis of BC by activating the SDF-1/CXCR4 axis.	(38)
	Co-cultured cell model of BC (MCF-7 and MDA-MB-231); murine BC model (MCF-7 and MDA-MB-231)	Leptin could promote BC growth and progression.	The absence of leptin receptor modified BC phenotype less aggressive in *vitro* and reduced the macrophage recruitment in *vivo*.	(39)
Adiponectin	Murine BC model	Adiponectin could accelerate the BC recurrence.	NA	(40)
	Cultured cell model of BC (MCF-7 and MDA-MB-231); murine BC model (MCF-7 and MDA-MB-231)	Adiponectin could inhibit ERα– BC growth and progression.	Adiponectin played an inhibitory effect on the growth and progression of ERα– BC cells in *vitro* and in *vivo*	(41)
	Obese BC patients	There was an association between adiponectin gene polymorphism, serum adiponectin level, and BC risk in obese postmenopausal women.	NA	(42)
	Obese BC patients	Polymorphism and promoter methylation of the adiponectin gene were associated with BC risk in obesity.	NA	(43)
Resistin	BC patients	Resistin might be a predictive marker in BC treatment.	NA	(44)
	Cultured cell model of BC (T47D, MCF-7 and MDA-MB-231)	Resistin could stimulate both ERα+ BC and TNBC progression.	NA	(45)
	Cultured cell model of BC (MCF-7, MDA-MB-231 and MCF-10A)	Resistin could promote BC metastasis.	Resistin induced BC cells EMT and stemness through both CAP1-dependent and CAP1-independent mechanisms.	(46)
	Cultured cell model of BC (MCF7, T47D, ZR-75-1, MDA-MB-231 and Hs-578 T); murine BC model (MDA-MB-231); zebrafish model	Resistin could facilitate BC progression by induction of EMT and stemness properties of BC cells.	Resistin facilitated BC progression *via* TLR4-mediated induction of mesenchymal phenotypes and stemness properties.	(47)
Visfatin	Cultured cell model of BC (MCF-7 and MDA-MB-231)	Visfatin could induce BC proliferation and inhibit apoptosis.	Extracellular Visfatin induced proliferation through ERK1/2 and AKT and inhibited apoptosis in BC cells.	(48)
SFRP5	Co-cultured cell model of BC (MCF-7 and MDA-MB-231)	SFRP5 could inhibit BC migration and invasion.	Adipocyte-derived SFRP5 inhibited BC cell migration and invasion through Wnt and EMT signaling pathways.	(49)

DIO, diet-induced obesity; BC, breast cancer; EMT, epithelial-to-mesenchymal transition; obASCs, obesity-altered adipose stem cells; PDX, patient-derived xenograft; TNBC, triple-negative breast cancer; EVs, extracellular vesicles; CSC, cancer stem cell; VEGFA, vascular endothelial growth factor A; CAP1, cyclase-associated protein 1; SFRP5, secreted frizzled-related protein 5; MDSCs, myeloid-derived suppressor cells; NA, not applicable.

#### Leptin

Among the diverse adipokines, leptin has been acknowledged as a key candidate molecule linking obesity and BC, due to its vicious function in obese-related BC growth and metastasis. Leptin, a 16 kDa protein hormone overexpressed in obesity, is a crucial adipokine that can regulate appetite satiety. Aberrant leptin signaling is a hallmark of obesity and has been recognized to influence BC biology within TME, showing the potential of serving as a biomarker for BC risk in overweight/obese women (50).

The disruption of apical polarity in mammary glands is a sign of BC onset. The normal breast tissue samples characterized by an elevated leptin/adiponectin transcriptional ratio for obesity showed the altered distribution of polarity markers at the apex, while leptin level in breast tissue increased with overweight and obesity (26). The elevated leptin disrupted epithelial polarity and promoted premalignant alterations of the mammary gland in obese mice involving the activation of the PI3K/Akt pathway, providing a molecular basis for early alterations in epithelial architecture during obesity-mediated cancer initiation (26). Mahbouli et al. detected the antioxidant responses in three human mammary epithelial cells and confirmed that different regulatory effects of leptin on oxidative status depend on the tumor status of different mammary epithelial cells (27). Obesity-related leptin secretion may aggravate the carcinogenic effect of obesity on BC through the regulation of the cellular oxidative state. Obesity alters the activity of adipocytes or stem cells to acquire tumorigenic properties, which are subsequently recruited into the TME with high levels of secreted leptin, leading to tumor growth through leptin-mediated pathways. By *in vitro* assay, Juárez-Cruz et al. established that leptin promoted the secretion of the extracellular matrix remodelers, MMP-2 and MMP-9, and invasion in a FAK and Src-dependent manner, strongly suggesting that leptin promoted the development of a more aggressive invasive phenotype in mammary cancer cells (28). This result was similar to the study of Haque et al. that leptin could induce cell viability, epithelial-to-mesenchymal transition (EMT), sphere-forming ability, and migration of ERα+ BC cell line (29). The effect was possibly mediated by inhibiting CCN5 signaling *via* activating JAK/AKT/STAT-pathway. Sabol et al. verified that obesity-altered adipose stem cells mediated EMT *in vitro* and promoted the tumor metastasis of the TNBC patient-derived xenograft (PDX) model (30). Leptin produced by obesity-altered adipose stem cells (obASCs) is a key factor for mediating tumor metastasis. Notably, leptin could be encapsulated in extracellular vesicles derived from obese adipose tissue, thereby mediating pro-tumoral activities and the malignancy phenotype of BC cells (31). It was confirmed that diet-induced obesity (DIO) mice exhibited reduced survival, increased systemic metabolic and inflammatory disorders, upregulated tumoral cancer stem cell (CSC)/EMT gene signature, and greater leptin signaling (32). In cell culture experiments, leptin enhanced mammosphere formation, and the pro-cancer effects including cell viability, migration, invasion, and CSC- and EMT-related gene expression. Up-regulation of leptin signaling could form a causal relationship with obesity-associated TNBC development by promoting CSC enrichment and EMT.

Leptin can directly remodel the TME by inducing metabolic changes in tumor cells and recruiting immune cells such as macrophages, CD8+ T cells, and myeloid-derived suppressor cells (MDSCs), which are capable of producing pro-inflammatory cytokines to maintain angiogenesis and tumor growth (51). Yadav et al. proved that VEGFA was up-regulated in macrophages after exposure to adipocytes through releasing leptin, representing a possible mechanism for the increased risk of BC progression in obese individuals (33). Evangelista et al. investigated the gene expression of leptin was negatively correlated with the infiltration of tumor-reactive CD8+ T cells in human TNBC tumors from obese patients when compared to non-obese (34). Besides, It was noticed that leptin could downregulate CD8+ T cell functions through activating STAT3-fatty acid β-oxidation (FAO) and inhibiting glycolysis in obese mice fed on a high-fat diet (HFD) (35). On the other hand, HFD-induced MDSCs participated in tumor growth facilitation by inhibiting lethal CD8+ T cells (36). This was involved in the complicated crosstalk that leptin facilitated the myeloid-derived suppressor cells (MDSCs) accumulation, while MDSCs down-regulated the leptin production. Leptin could also drive the tumor to escape from immune attacks by enhancing fatty acid oxidation and tumor resistance to NK cell lysis *via* PGC-1 activation, thus showing a therapeutic clue for BC by blocking leptin (37). Duan et al. indicated that higher leptin receptor expression was related to increased malignancy and bone metastasis incidence in BC patients (38). The following *in vitro* assay further confirmed the role of leptin in promoting BC cell migration and invasion, *via* the SDF-1/CXCR4 axis activated by leptin. The absence of leptin receptor modified BC phenotype less aggressive *in vitro* and reduced the macrophage recruitment *in vivo*, proposing an innovative role of leptin receptor in modulating BC features (39).

Overall, leptin is emerging as the most important molecular mediator of the obesity-BC axis. The overactive leptin network interacts with BC cells directly or with different components in TME indirectly, thus participating in multiple steps of BC initiation and progression. Totally, the therapy targeting leptin deserves a deep evaluation in BC risk control and management for improving clinical outcomes and reducing mortality.

#### Adiponectin

Another adipokine that plays a critical role in obesity-associated BC is adiponectin with a molecular weight of 28-30 kDa, which is mainly secreted by adipocytes (52). There has been sufficient literature to prove the important role of adiponectin in the pathogenesis of obesity-related diseases (52). Ecker et al. observed that serum levels of adiponectin decreased in a diet-induced obese mouse model, which was negatively correlated with obesity and accelerated the BC recurrence (40). Mauro et al. certified the inhibitory effect of adiponectin on the growth and progression of ERα– BC cells *in vitro* and *in vivo*. While in ERα+ BC cells, low adiponectin levels, similar to the circulating concentration of adiponectin in obese women, acted as a stimulator for their growth and progression (41). Based on the above observations, higher doses of adiponectin and an appropriate combination of anti-estrogen therapy should be administered for ERα+ BC obese patients.

There are a variety of single-nucleotide polymorphisms in the adiponectin gene. Mahmoud et al. confirmed that there was an association between adiponectin gene polymorphism, serum adiponectin level, and the risk of BC in obese and overweight postmenopausal women (42). Besides, Pasha et al. simultaneously studied the genetic and epigenetic changes of the serum level of adiponectin gene and uncovered that polymorphism and promoter methylation of the adiponectin gene were associated with BC risk in obesity (43). A new non-invasive biomarker designed by evaluating the methylation status of the adiponectin gene promoter can be produced and used as an encouraging tool for early detection of BC.

Decreasing levels of adiponectin in the obese woman may reduce the beneficial effects of adiponectin on BC, including inhibiting BC cell proliferation or stimulating apoptosis of BC cells. Novel therapeutic approaches toward adiponectin could be of great significance in the prevention or treatment of BC for obese women.

#### Resistin

Resistin, a 12.5 kDa protein encoded by the RETN gene, is related to obesity, inflammation, and various cancer type, including BC. Based on the anthropometric data and parameters, Patrício et al. indicated that resistin and glucose, as well as age and BMI, could be used as a powerful biomarker of BC (53). Wang et al. identified high levels of resistin in BC patients in Chinese Han women, and resistin might be a predictive marker in BC treatment (44). They also proved the association between polymorphisms of the RETN gene and susceptibility for BC.

Growing evidence has indicated that resistin is upregulated in BC patients, yet the mechanisms of resistin on adjusting BC behavior during obesity are still largely unknown. Rosendahl et al. suggested that resistin significantly stimulated both ERα+ BC and TNBC cell progression by utilizing the most extensively used adipocyte model *in vitro*, which could be further strengthened under obesity-related metabolic conditions (45). CAP1, as a newly identified resistin receptor, was expressed across a large panel of BC cell lines and primary BC tumors, and high expression of CAP1 was associated with poorer tumor characteristics, higher histological grades, and impaired prognosis among BC patients, highlighting the potential role of CAP1 concerning BC outcome. Besides, Avtanski et al. pointed out that adipokine resistin could enhance the metastatic potential of BC cells *via* inducing EMT and stemness *in vitro*, partially mediating by CAP1 (46). Similarly, Wang et al. observed that high expression of serum resistin in BC patients was positively associated with tumor stage, size, lymph node metastasis, and poor patient survival (47). Their study verified that obesity-related resistin facilitated BC progression by induction of EMT and stemness properties of BC cells *via* activating TLR4/NF-κB/STAT3 signaling in animal models of BC tumorigenesis and metastasis. Gao et al. confirmed that obesity elevated the expression of adipocytic TAZ/Resistin (a functional downstream target of TAZ), and facilitated tumorigenesis *in vitro* and *in vivo*. They also found that the resistin expression was strongly related to adipocytic TAZ and advanced clinical stage in TNBC samples (54).

#### Other Adipokines

Visfatin is an adipokine and proinflammatory cytokine secreted by adipocytes, macrophages, and inflamed endothelial tissue and is dramatically increased in obese BC patients. Elevated levels of visfatin may promote the development of BC and reduce the effectiveness of treatment in BC patients (48). Gholinejad et al. discovered that Extracellular Visfatin induces BC cell proliferation through activation of the AKT/PI3K and ERK/MAPK signaling pathways and inhibits apoptosis in these BC cells (48). The role of visfatin in the development of BC further confirms the relationship between obesity and BC.

SFRP5 is a novel adipokine with anti-inflammatory properties and is related to obesity. The plasma level of SFRP5 is significantly reduced in obese patients and SFRP5 expression is also decreased in various tumor tissues (49). Zhou et al. revealed that in BC patients, SFRP5 is negatively correlated with BMI, lymph node metastasis, infiltration, TNM stage and higher Ki67 expression, and elevated SFRP5 levels were connected with the improved prognosis of BC patients (49). In addition, they detected reduced SFRP5 level in the obesity-induced hypertrophic adipocyte model, and the hypertrophic adipocytes augment BC cell invasion and migration through inhibiting SFRP5 expression. The promotion effect of hypertrophic adipocytes was reversed by the addition of SFRP5 *via* downregulating Wnt and EMT signaling pathways. SFRP5 is a vital adipokine that could mediate the crosstalk between obesity and BC metastasis and new therapy by promoting SFRP5 expression in the adipose microenvironment might be an effective way in preventing BC metastasis.

### Insulin and IGF

In obesity, the expansion of adipose tissue can lead to a chronic inflammatory state contributing to the elevated levels of circulating insulin, IGF, and obesity-related insulin resistance, thus creating a more favorable TME for carcinogenesis (4). Hyperinsulinemia has become a research hotspot as a potential mediator for the growth of obesity-related BC, and the increased plasma levels of insulin independently predict the increased risk and mortality in obesity-related tumors, including BC and several other tumor types (17, 55) Obesity-related hyperinsulinemia could lead to the abnormal insulin signaling pathway, which may affect the expression and localization of insulin receptor (InsR), in BC (56). According to the study by Swedish scholars, obese patients with nuclear InsR–/ER– had the worst prognosis of all 900 patients with primary invasive BC (57).

Insulin can act synergistically with inflammation to promote BC growth and metastasis (58). Insulin could increase the expression of VEGFA in macrophages and have pro-angiogenic effects *in vitro* endothelial tube formation assay (33). Rabin-Court et al. validated that the obesity-driven elevated insulin could upregulate mitochondrial glucose oxidation in obesity-associated tumor cell lines (BC, colon cancer, and prostate cancer cells) correlated with a dose-dependent increase in cell division (59). Rodriguez-Monterrosas et al. proposed that insulin, by activating insulin-like growth factor 1 (IGF-1) receptor, induced the proliferation, migration, invasion, and an enhanced MMP-9 secretion in MDA-MB-231 cells pretreated with linoleic acid *in vitro*, strongly suggesting the important role of insulin signaling in BC invasion and metastasis (60). Insulin resistance, associated with hyperinsulinemia, hypertension, and impaired glucose tolerance, has been considered as the underlying cause of the relationship between obesity and high BC risk, prognosis, and survival. Through the more accurate assessment and appropriate stratification of patients by BMI and menopausal status in a prospective study, Luque et al. revealed a clear association between BC presence and higher insulin resistance in overweight/obese premenopausal women (61).

IGF-1 is an insulin-related hormone that is involved in a variety of physiological and pathological processes, including cell proliferation and differentiation, tumor growth, and metastasis. IGF-1 has been indicated as an endocrine BC risk factor. Ecker et al. utilized a genetically engineered mouse model that outlined the physiological characteristics of obese patients and showed that HFD obese mice exhibited hyperinsulinemia, increased IGF-1 levels, and accelerated BC recurrence when compared with the control mice, suggesting that the insulin/IGF-1 signaling pathway is a potential mediator of the relationship between obesity and BC recurrence (40). Tong et al. found that high IGF-1 was related to an impaired 4-year recurrence-free survival (RFS) for overweight patients in a retrospective study of HER2+ BC patients (62). It was the first and largest study to prove the significant interaction between IGF-1 and BMI in predicting RFS and OS in HER2+ BC patients. Hillers et al. injected the mixed adipocyte-derived stem cells (ASCs) which were isolated from HFD fed mice or the control diet with BC cells into the mammary glands of lean mice and observed that obese mouse ASCs induced an invasive phenotype of BC cells by increasing the expression of IGF-1 (63).

Thus, obesity is recognized to be a state of chronic inflammation characterized by elevated circulating levels of pro-inflammatory mediators, including IL-6, C-reactive protein (CRP), CC chemokine ligand 2 (CCL2), TNF-α, and disordered non-coding RNA, which function in the BC cell proliferation, invasion, and migration in a paracrine manner. Meanwhile, inflammation is a common symptom in both obesity and BC and acts as a direct bridge between the two. Besides, the inflammation milieu can even interact and collaborate with hyperglycemia, adipokines, hormone-associated aromatase alteration, thus creating an extraordinarily complex obesity-inflammation axis for supporting BC progression. Novel therapeutic interventions targeting and controlling inflammation would be of great value in obesity-related BC.

### Sex Hormone

Most of the BC are hormone-dependent tumors, and the increased exposure to sex hormones may facilitate the development of BC. It was reported that elevated steroid hormone levels in obesity increased the mortality in hormone receptor-positive BC patients (64). In addition, increased estrogen levels were associated with obesity and independently correlated with an elevated risk of BC (65). Adipose tissue converts androstenedione to estrone by aromatase of stromal cells to produce estrogen after menopause. A randomized, placebo-controlled trial confirmed that the expression of aromatase, the rate-limiting enzyme in estrogen biosynthesis, was increased in the breast tissue of obese patients, which led to an elevated risk of HR+ BC in obese postmenopausal women and docosahexaenoic acid could reduce the expression of aromatase (66). Subbaramaiah et al. pointed out that prostaglandin E2 down-regulated sirtuin1 leading to an increase of aromatase in breast tissues of obese women, providing novel insights into the obesity-BC connection (67).

Sabol et al. showed that obASCs promoted ER+ BC tumor growth through estrogen signaling *in vivo*, while for metastasis, the promotion effect of obASCs was not entirely estrogen-dependent (68). Zhao et al. found that in the breast tissue of premenopausal obese women, the expression of genes related to estrogen (such as ESR1 and GATA3) and genes involved in cell growth and protein synthesis (such as RPS6KB1) was significantly reduced in breast tissue. In postmenopausal obese women, the inflammatory molecule PTGS2, ESR1 target gene TFF1, and cell cycle G1/S checkpoint gene CCND1 were elevated (69). The differential expression of these genes may help to explain the difference in BC-promoting effects of estrogen in obese women according to menopausal status. Qureshi et al. showed that the postmenopausal estrone and premenopausal 17β-estradiol played opposing roles in BC-promoting effects of obesity. Estrone is pro-inflammatory in contrast to the anti-inflammatory actions of 17β-estradiol and increases with obesity, and stimulates the expansion of tumor-initiating stem-like cells in ER+ BC to drive rapid BC growth *in vivo* (70). Understanding the hormonal environment in tumor tissue may be crucial to elucidating the BC etiology and improving patient outcomes.

The excessive fat accumulation, chronic low-grade inflammation, and numerous pro- and anti-inflammatory factors caused by obesity are closely related to elevated estrogen/aromatase expression and activity in post-menopausal women (71). These changes are determined as the underlying force of obesity in promoting the occurrence of postmenopausal BC (72). In general, aromatase and estrogen may still be key factors in the link between obesity and poor prognosis in ERα positive, post-menopausal BC patients. The aromatase overexpression in dysfunctional obesity states indicates its potential pharmacologic target for BC prevention.

As important inflammatory mediators, interleukin (IL)-6, tumor necrosis factor (TNF)-α, CCL2, cyclooxygenase-2 (COX-2), and prostaglandin E2 (PGE2) catalyzed from COX-2 participate in the induction of aromatase expression in breast adipose tissue. In obese mice and subcutaneous adipose tissue from obese women, CCL2 was increased in breast adipose tissue and enhanced the glucocorticoid-mediated CYP19A1 transcription, thus promoting the pro-inflammatory milieu and aromatase expression under obesity condition (73). Hypoxia-inducible factor 1α (HIF-1α) was a dominant regulator of oxygen homeostasis and could stimulate aromatase and CREB1 expression in response to tumor-derived and obesity-associated pro-inflammatory mediator PGE2 (74). Zahid et al. supported that the obesity-associated increased the aromatase expression in human breast tissue, at least partially due to the increased *in situ* expression of aromatase (75). They also confirmed the involved mechanism by which leptin could regulate aromatase *via* the activation of the p53-HIF-1α/PKM2 axis. TNF-α was a key driver of aromatase gene expression. Obesity was related to the enhanced TNF-α and reduction in local IL-10, which mediated the aromatase and estrogen biosynthesis in mammary adipose tissue, providing novel insight for prevention strategy of post-menopausal obesity-associated BC (76).

In the mammary gland and visceral fat of obese mice, the increase of aromatase mRNA and activity was paralleled with the increase of TNF-α, IL-1β, and COX-2. These pro-inflammatory mediators in turn promoted the induction of aromatase (77). The increase in fat mass and aromatase may be responsible for the increased risk of HR+ BC in postmenopausal obese women. High-sugar/fat diet increased the levels of pro-inflammatory mediators CCL2, IL-6, COX-2, and PGE2 in breast tissue, accompanied by the formation of breast coronal structure and the up-regulated biosynthesis of aromatase/estrogen (78). It showed that the obesogenic diet accelerated BC carcinogenesis in a COX-2-dependent manner. By *in vitro* verification, Bowers et al. showed that obesity-induced systemic IL-6 indirectly increased the aromatase expression derived from pre-adipocyte through augmented BC cell PGE2 production, which resulted in a subsequent increase in BC cell ERα activity and proliferation (79). It suggested that obesity IL-6 might be a potential mechanism to enhance the postmenopausal, hormone-responsive BC progression *via* an elevated local aromatase expression.

Therefore, local production of inflammatory mediators surrounding adipose tissue and in the tumor, and estrogen increase, which is thought to establish a carcinogenesis-promoting microenvironment and further drive tumor growth by significantly increasing the role of aromatase. Obesity-related aromatase increases in adipose tissue and the whole body system, while hormone therapy lifestyle interventions including weight management and may reduce BC risk by decreasing levels of related hormones and aromatase.

### Chronic Inflammation

The relationship between obesity and BC is ascribed to diverse factors, while the chronic low-grade inflammation accompanied by obesity is the closest link between them. Recently, the significant role of white adipose tissue (WAT) inflammation in the progression and metastasis of obesity-related BC has been gradually recognized (66, 80). The results of a retrospective study showed that breast WAT inflammation is usually generated in overweight/obese BC women and may be related to abnormal circulatory indicators related to metabolic syndrome (81). Previous studies have confirmed the systemically BC promotion effect of adipose inflammation *via* circulating pro-inflammatory cytokines, such as IL-6 and TNF-α (82). Since BC is located in a fat-rich environment, recent studies have mainly focused on the local effects of inflamed adipose tissue, including the impact of cytokine levels in mammary adipose tissue on BC (83). A study of Australian scholars indicated that obesity reduced the local IL-10 level in the mammary fat pad of ovariectomized mice, and the reduced IL-10 enhanced the expression of aromatase in mammary fad in ovariectomized mice, contributing to BC development and progression (76).

In obesity, breast adipose tissue induces inflammation by increasing the expression of pro-inflammatory cytokines and the recruitment of macrophages, and the elevated pro-inflammatory cytokines, in turn, upregulated genes and signaling pathways that contribute to breast inflammation and BC progression (84, 85). Notably, the crown-like structures (CLSs), constituted by necrotic and dying adipocytes encircled by macrophages in adipose microenvironments, are a histologic hallmark for the pro-inflammatory process (86). Studies have shown that the CLSs of the breast are more frequently detected among obese compared to non-obese BC patients and high CLS-densities are independently associated with an increased BC risk (87). CLS-related pro-inflammatory behaviors are believed to increase the risk of worse BC prognosis in obese or post-menopausal patients. As confirmed by Maliniak et al., there was an intensive positive association between BMI and CLSs from breast adipose tissue in non-tumor tissue, which was independent of race (87). Importantly, the adipocyte-macrophage crosstalk in obesity-related BC mainly involves excessive inflammatory cytokines secretion (TNF-α, IL-1β, IL-6, and PGE2), TSC1-TSC2 complex-mTOR, insulin resistance, endoplasmic reticulum stress, and increased levels of aromatase activity and estrogen production. For instance, in both dietary and genetic models of obesity, Subbaramaiah et al. proved observed the presence of CLSs with a typical structural feature, which was related to NF-kB activation, increased pro-inflammatory cytokines, and the elevated expression and activity of aromatase in the mammary gland (77). Moreover, large epidemiologic studies have demonstrated that obesity positively contributes to the formation of CLSs and their association with clinical BC outcomes (88). Therefore, targeting CLSs in breast adipose tissue emerges as a prominent therapeutic strategy, and the tests of body adipose tissue composition and inhibition of inflammation state will be of value to direct combinatorial approaches.

Wilcz-Villega et al. proved that conditioned medium of macrophages derived from human healthy donors facilitated the acquisition of malignant traits (such as anchorage-independent growth and invasiveness) in mammary epithelial cells in 3D culture, which was mediated by IKKe/TBK1 kinases and the serine biosynthesis pathway (89). Tiwari et al. proposed a pro-inflammatory metabolically activated phenotype (MMe) macrophage reprogrammed by obesity that could promote tumorigenesis, which is different from the pro-inflammatory M1 macrophage that antagonized tumorigenesis (90). They showed that MMe macrophages represented the main macrophage phenotype in mammary fat of obese women and mice. MMe macrophages secreted IL-6 in a NOX2-dependent manner through combining with GP130 on TNBC cells to promote stem-like properties and tumorigenesis during obesity. Besides, Kolb et al. identified that obesity increased tumor-infiltrating macrophages with NLRC4/IL-1β-dependent upregulation of angiopoietin-like 4, contributing to increased angiogenesis and BC progression in the obese mouse tumor (91).

The influence of obesity-induced inflammation and hormone production on tumorigenesis is determined by the menopausal status. Cranford et al. found that HFD induced inflammation was correlated with increased tumorigenesis in an ovariectomized mouse model of premenopausal hormone receptor-positive BC, but had no significant effect in postmenopausal mice (92). Certain micro-RNAs (miRs) that participated in the regulation of inflammation are known as inflamma-miRs. A cohort study of Tunisian patients reported that chronic inflammation in obese BC patients was associated with aggressive BC by inducing overexpression of oncomiRs (such as miR-21 and miR146a) and reducing the expression of tumor suppressor miRs (such as miR-34a) (93). More comprehensive mechanism studies connecting obesity-driven inflammation with BC are needed for more potential therapeutic strategies of BC. In total, obesity often leads to insulin resistance, which can cause compensatory hyperinsulinemia. Adipocyte dysfunction in the context of obesity is the basis of insulin resistance and chronic inflammation, which can lead to the development and progression of BC. Moreover, the cross binding of insulin to the IGF-1 receptor expressed on BC cells stimulates the proliferation of BC cells. Therefore, insulin and IGF-1 have been identified as BC promoters that activate many pathways that drive aggressive BC biology. The favorable insulin signaling control will optimize BC risk prevention and BC survival.

## The Evolving Role of Obesity in Breast Cancer Therapy

### Surgery

Generally, obesity is thought to be a risk factor in surgery procedures due to the increased incidence of complications. Patients with obesity are at increased risk of developing complications of varying degrees [such as infection (94, 95) seroma formation (96), lymphedema (97), flap thrombosis/necrosis (98), and delayed breast cellulitis (99)] after mastectomy alone or in combination with autologous/implant-based immediate breast reconstruction (100). Garland et al. indicated that increasing obesity progressively increased the postoperative complication rates even without BC reconstruction (101). Obesity is indispensable information of preoperative consultation and appropriate risk stratification for BC patients. Most of the surviving patients who undergoing BC surgery suffer from persistent pain, which greatly reduced their quality of life. Ding et al. reported that BMI might be positively correlated with the risk of persistent pain after BC surgery (102).

With the rising prevalence of obesity, breast reconstruction in obese patients is becoming the norm rather than the exception nowadays. In plastic surgery studies comparing autologous and prosthetic reconstruction between obese and nonobese patients, it found an increased complication rate and a decreased satisfaction rate in the obese group (103). Consistently, in a retrospective study, the obese patients with BC reconstruction after mastectomy exhibited increased complications and failure rates compared with the normal population, while reconstruction with free tissue transfer from the abdomen presented more satisfactory outcomes and decreased complications than prosthetic reconstruction (103). Chang et al. also proved the safety of autologous breast reconstruction in obese patients (104). These results meant that obesity was not contraindicant and might be an excellent option for reconstruction in obese patients. For obese women with surgical risk factors for prosthetic breast reconstruction, surgical modification can also reduce the occurrence of perioperative complications (105). Therefore, the personalized assessment of preoperative risk, intraoperative techniques, and postoperative management are essential to maximize prognosis and reduce complications for obese patients.

### Radiotherapy

Post-surgery adjuvant RT significantly reduces the local recurrence rate and ameliorates the clinical outcomes of BC patients. RT-caused adverse responses, mainly including skin toxicity, pain, lymphedema, and telangiectasia, are caused by RT damage to the surrounding normal tissue and negatively impact the overall quality of patient life. Cutaneous inflammation and toxicity may be a major source of RT-related pain in BC patients. The inflammatory biomarker CRP is an indicator of RT-related pain in BC patients. Intriguingly, Lee et al. firstly proposed that obesity was a correlative factor, as more increased risk of RT-related pain appeared in obese patients (pre-RT CRP ≥ 10 mg/L) than those non-obese patients (pre-RT CRP < 10 mg/L) (106). In BC patients with RT therapy, Hu et al. also pointed out the risk of obesity-related to Grade 4+ skin toxicity and other late effects (107). Obesity-induced metabolism abnormalities are crucial for aggravating RT resistance and poor prognosis of BC. In a retrospective study with BMBC patients treated with RT, McCall et al. confirmed that BMI negatively affected OS and local control, showing the significance of BMI for prognosis and clinical trial design by using as a stratification (108). The *in vitro* assay of Sabol et al. evaluated the effects of obesity-altered ASCs on ER+ BC cell response to RT (109). They concluded that obesity could alter the ASCs phenotype to confer undesired RT resistance *via* enhanced secretion of leptin by ASCs, promoted the production of IL-6, and activated Notch pathways in these BC cells. Thus, it posed evidence of obesity-related paracrine effect in shaping obese BC prognosis.

### Chemotherapy

Emerging clinical studies have shown that obesity is able to hurt chemotherapy efficacy, leading to a reduced likelihood of achieving pathological complete response (pCR) in obese and overweight patients. Interestingly, multi-agent regimens also enhance the risk of gaining weight during chemotherapy, and this weight gain might reduce the effectiveness of treatment. Obesity-mediated drug resistance can be achieved by altering drug pharmacokinetics, formulating chronic inflammation, and promoting tumor-related adipocyte adipokine secretion (110). By using a co-culture system to grow BC cells with primary mammary adipocytes isolated from lean and obese patients, Lehuédé et al. found that these human mammary adipocytes induced chemoresistance in BC cells, which was amplified by obesity (111). Adipocytes induced a multidrug-resistant phenotype in BC cells, as the level of major vault protein was increased to promote doxorubicin (DOX) nuclear efflux. Liu et al. showed that resistin treatment could induce autophagy to decrease the DOX-induced BC cell apoptosis *in vitro*, suggesting that upregulated levels of resistin conferred DOX resistance in BC therapy (112). Therefore, targeting resistin might be a novel strategy to improve chemoresistance. Mentoor et al. found that DIO weakened the DOX efficacy in a breast tumor-bearing mouse model (113). They found that both the expression level of leptin and resistin were significantly increased in the HFD group treated with DOX, confirming obesity conditions induced the changes in tissue fatty acid composition to further reduce the therapeutic effect of DOX.

Obesity is also associated with some side effects of chemotherapy. For young BC patients before menopause, adjuvant chemotherapy may cause interrupted menstruation and premature menopause, which may damage their life quality. Yeo et al. led a study of 280 young Chinese women with premenopausal BC who received adjuvant chemotherapy for 3 to 10 years, concluding that overweight/obesity was associated with more severe menopausal symptoms (114). To determine the relationship between obese BC patients and febrile neutropenia, Collins et al. conducted a single-center, retrospective chart review (115). The results indicated that obese patients had no increased risk of febrile neutropenia, but the threshold for febrile neutropenia was lower and required more antibiotics after chemotherapy. It is worth noting that up to 90% of BC patients experience cancer-related fatigue (CRF), which is considered the most durable and painful physical injury after treatment. Inglis et al. evaluated the obesity impact on CRF in BC patients and found that obese patients underwent higher CRF from before chemotherapy to 6 months after chemotherapy (116). Active measures related to weight loss interventions and diet changes may improve the CRF degrees of obese BC patients pre- and post-chemotherapy.

Aldo-keto reductase (AKR) is a supergene family which comprises 14 families and more than 40 members. AKR enzymes are NADPH-dependent oxidoreductases that can interconvert carbonyl groups with alcohols. But recent studies have shown that AKR1 takes part in the malignant transformation of some human tumors, such as BC, and as well as in the resistance to cancer treatment (1). AKR1C3, belonging to the AKR family, may play an important role in the development of hormone-dependent or hormone-independent BC. It is known that AKR1C3 is abundantly expressed in BC and is associated with a worse prognosis. AKR1C3 is also related to DOX resistance in human BC (2). Byrns et al. confirmed that the expression of AKR1C3 in MCF-7 cells resulted in the elevated ratio of 17β-estradiol: progesterone, conferring a proliferative preponderance to BC cells (3). AKR and carbonyl reductases (CBR) enzymes can metabolize a variety of drugs and obese individuals can express high levels of these enzymes. It seems that AKR and CBR enzymes might play an important role in obesity-induced BC drug resistance. Sheng et al. demonstrated that obesity was associated with higher adipose tissue expression levels of AKR isoenzymes, including AKR1C1, AKR1C2, and AKR1C3, prompting the inactivation of anthracyclines, such as daunorubicin, by conducting mouse experiments and human tissue verification for the first time (4). These alterations may lead to the local reduction of efficacy of chemotherapy and result in BC chemotherapy resistance.

The effect of obesity on BC patients receiving neoadjuvant chemotherapy (NAC) is still a matter of debate. Some studies have shown that obese and overweight patients are less likely to achieve NAC pCR, while others have found no significant difference in pCR between obese and non-obese women. Karatas et al. assessed BMI and pCR to NAC and revealed that obesity was associated with lower pCR to NAC and lower OS, which may be caused by the reduction of therapeutic dose in this group of patients due to obesity-induced chemotherapeutic resistance (117). The NeoALTTO trial showed that obesity and overweight were associated with a decreased chance of HER2+ luminal BC patients obtaining pCR (118). Notably, there was no differential effect observed between BMI and pCR in HR– cases, leading to a statistically significant interaction between BMI and HR status. Obese BC patients are known to have a lower pCR rate, and more aggressive, dose-free chemotherapy combinations could be considered for better efficacy. Farr et al. retrospectively analyzed the obesity-related effects on pCR and survival (119). The main finding was that obese women who received a full unbounded dose of anthracycline-taxane-based NAC increased pCR and benefited progression-free survival, which might also lead to increased dose intensity associated with improved efficacy and toxicity. Méndez-Hernández analyzed the serum and tissue samples of primary BC patients in Mexican that received neoadjuvant therapy and confirmed that the polymorphisms of Leptin (LEP rs7799039) and adiponectin (ADIPOQ rs1501299) were risk-contributing factors in overweight/obesity patients (120). These genotypes influenced the response to chemotherapy, indicating that the obese microenvironment was more inclined to tumor progression and drug resistance by defining TME.

The American Society of Clinical Oncology (ASCO) clinical practice guidelines published a study on the appropriate chemotherapy dose for obese adult cancer patients in 2012 (121). Morrison et al. added the evidence supporting the use of actual weight-based dosages in accordance with the 2012 ASCO guideline for appropriate chemotherapy dosing, including in older cancer patients (122). Dose adjustment is a feasible strategy to potentially improve long-term survival in obese patients without increased toxicity. A prospective PANTHER study also showed that customized, dose-intensive epirubicin/cyclophosphamide and docetaxel were associated with improved BC relapse-free survival in obese patients compared to standard care, but not in non-obese patients (123). Natural phytochemicals can affect BC proliferation and metastasis signaling networks by regulating chronic inflammation related to excess, and sensitize the efficacy of chemotherapy drugs on BC cells (124). Some novel combinations of phytochemicals, developmental agents and/or chemotherapeutic agents will facilitate the achievement of effective solutions that expand and enhance multi-tiered intervention strategies for BC prevention and treatment. A series of further studies with a larger sample will enable us to better understand the dynamic changes of obesity to BC chemotherapy and other adjuvant treatments synergistically.

### Endocrine Therapy

Since approximately 75% of BC express two hormone receptors; ER and/or PR, adjuvant ET is used in the treatment of most BC patients, including anti-estrogen therapy or suppression of estrogen production (125). The standard therapy for most ER+ BC patients includes selective ER modulators and aromatase inhibitors (AIs), which can counteract the tumorous on ER activity or suppress the adipose tissue to aromatize androgens into estrogen. Obesity increases the levels of circulating sex hormones, including estrogen, and is associated with a higher risk of ER+ BC and ET side effects. This will cause ET to be less effective in obese women with BC. AIs are the treatment of choice for some women with ER+ BC, but there are reports that tamoxifen (Tx) may be more effective for obese women compared with AIs (126, 127). Zewenghiel et al. investigated the effect of BMI on ET efficacy in postmenopausal women with metastatic HR+ BC and showed that fulvestrant and AIs had no difference in the efficacy among normal, overweight, and obese women (128). Regarding the relationship between BMI and decreased efficacy of AIs, the existing results are still controversial. Obesity is associated with a reduced risk of fracture in healthy postmenopausal women, but for patients with early BC treated with AI, obesity may have the opposite effect. One possible mechanism for this was that AIs inhibited estrogen production, resulting in a loss of protection against fragility-related fractures (129). Obesity may become an additional parameter for clinical decisions to use bisphosphonates or denosumab to reduce fracture risk in ET patients with AI.

The mechanism of obesity in promoting ET resistance is complex and has been well concerned. In the athymic nude mice, the obesogenic diet could induce obesity, glucose tolerance, and hyperinsulinemia, and insulin resistance, which further mediated letrozole resistance. This effect might partially be attributed to the higher aromatase expression in the adipose tissue with obesity (130). In order to understand the mechanism of the weakened effect of ET on BC cells in the presence of the adipocyte secretome, mature adipocytes were co-cultured with BC cells and treated with ET (Tx, fulvestrant, Showing that the interplay between the adipocyte group and cells together played an important and complex role in ET resistance (131). Hagen et al. quantified non-adherence and discontinuation to ET in post-menopausal women with BC and found that overweight and obesity were time-dependent risk factors predictive for ET termination (125). Maintaining a normal BMI can improve ET compliance in postmenopausal women with BC. Wellberg et al. demonstrated that obesity and excess energy shaped a tumor environment characterized by ET resistance, and identified the participation of FGFR1 signaling in obesity-related BC progression (132). In the co-culture system of MCF7 cells and human adipocytes exposed to high Glucose, adipocyte-derived IL-8 mediated the enhanced connective tissue growth factor (CTGF) mRNA and reduced tamoxifen responsiveness of BC cells (133). Moreover, targeting IL-8 in the TME could not only reduce the inflammatory state but also indirectly modulate CTGF, thus improving the effectiveness of tamoxifen treatment in BC. Morgan et al. cultured the patient-derived stroma in an organotypic breast model *in vitro* and found that MCF7-derived ducts co-cultured with obese stromal cells possessed higher maximal aromatization-induced ER transactivation and reduced anastrozole sensitivity (134). This suggests that breast adipose stromal cells from obese women decrease AI sensitivity and supported obesity as a therapeutic for ER+ BC patients. Strong et al. suggested that ER+ BC cells were responsive to the obASCs with enhanced growth and EMT during direct co-culture mode, whereas lnASCs were unable to increase ER+ BC growth (135). The mechanism validation showed the obASC-derived leptin was a key molecule to drive BC tumorigenicity and potential ET therapy. More intriguingly, compare to normal control, the plasma exosomes from obese women significantly promoted the BC cell proliferation, migration, invasion, and resistance to tamoxifen, indicating that the obese circulating exosome was a potential mediator of adipose tissue involved in tamoxifen resistance.

Specific adipokines could reduce the efficacy of ET in the treatment of overweight/obesity. Bougaret et al. studied whether mature adipocytes and their secretions from adipose stem cells of normal-weight (MA20) or obese (MA30) women, could influence the effects of Tx (136). In a co-culture 3D model, the anti-proliferative effect of Tx on MCF-7 BC cells was counteracted by MA30. Besides, leptin, IL-6, and TNF-α could decrease the anti-proliferative efficacy of the active metabolite 4-hydroxytamoxifen of Tx. Especially, it is worth noting that obesity-related leptin has been confirmed to interfere with the endocrine therapy outcomes in BC patients, such as tamoxifen. Obese patients secrete a large amount of leptin to exert proliferative, mitotic, anti-apoptotic, and pro-inflammatory activities, thus may have antagonistic effects on the treatment of Tx (137). For example, the *in vitro* leptin administration could result in promoted tamoxifen-resistance *via* leptin/Ob-Rb/STAT3 pathway by regulating the apoptosis-related genes BCL2 and WWOX in HER2-overexpressing BC cells (138). Besides, Qian et al. found that leptin-mediated tamoxifen resistance of BC was correlated with the activation of ERK1/2 and STAT3 signaling and overexpression of cyclin D1 in MCF-7 BC cells by binding to ObRb (139). Similarly, Gelsomino et al. found a role of obesity-related leptin in sustaining AIs resistance that leptin signaling boosted the AIs resistant BC cell growth and macrophage activation (140). Therefore, the hyperactive leptin signaling network influences the BC through direct effects on tumor cells or indirect impacts on different components of the TME (141). This evidence highlights the clinical value of targeting leptin in improving the hormone therapy for BC in obese BC patients. In particular, identifying specific adipokine levels could help individualize the management of overweight BC patients. The efficacy of leptin-targeted drugs deserves in-depth exploration in the individualized management of overweight BC patients to improve clinical outcomes and decrease mortality. Furthermore, because obesity loses an equilibrium in the energy metabolism and has a modifiable nature, change body weight index, adjust feature of fat metabolism, reduce adipose factor maladjusted, have benefited greatly to performing cancer prevention and cure.

### Immunotherapy

Obesity has become a risk factor for an attenuated anti-tumor immune landscape. Obesity may up-regulate the pro-inflammatory adipokines and down-regulate the anti-inflammatory adipokines expression levels, leading to an excessive adipokine secretion and a persistent immune imbalance accompanied by a chronic inflammatory. Importantly, the characteristics and functions of immune cells have undergone largely impaired biological changes with the obese state (142). The tumor-killing effector cells (CD8+ T) were suppressed and the immunosuppressor cells (MDSCs/M2) were over-activated to drive their recruitment and suppressive capacity, which could be mediated by obesity-related molecular markers, such as IL-6, CRP, leptin, IL-1β.

For instance, in elderly mice given systemic anti-tumor immunotherapy, obesity caused a fatal cytokine storm, which increased M1 macrophage polarization, and proinflammatory TNF-α and IL-6 release, leading to a reduced anti-tumor effect and low survival rate (143). PD-L1 has been shown to be overexpressed BC than in normal breast tissue and is closely associated with tumor immune surveillance and prognosis. Wang et al. found that obesity or metabolic syndrome-related M1 macrophages up-regulated PD-L1 expression in TNBC by partially secreting IL-6 in a JAK/STAT-dependent pathway, which in turn affected immunotherapy targeting the PD-L1/PD-1 axis *in vitro* (144). Gibson et al. firstly identified the effect of obesity on MDSC-mediated immunotherapy resistance in a BC mouse tumor model and found that obesity could lead to the accumulation of FasL+ granulocytic MDSCs, thus promoting apoptosis of tumor-Infiltrating CD8 T cells and immunotherapy (adenovirus encoding TRAIL + CpG) resistance in BC (145). It posed a novel pathway of MDSC-related resistance and its disruption may improve immunotherapy outcomes in patients with BC and obesity.

Overweight and obese women with BC are prone to acquire worse clinical outcomes with antibody-based immunotherapy, which has been confirmed by some clinical studies. The inherent associations potentially operating to link obesity and prognosis in BC are complex. The adipocyte secretion-associated alteration in obesity, including the changes in the ratio of leptin to adiponectin, the expression levels of pro-inflammatory cytokines, IGFs, and estrogen, may be the mechanisms potentially leading to BC immunotherapy resistance. For example, Ado-trastuzumab emtansine (T-DM1) is a novel antibody-drug conjugate suitable for the treatment of HER2+ BC. In obese patients receiving T-DM1 might require more treatment modifications secondary to adverse events compared to non-obese patients (146). In a cohort of HER2- metastatic BC patients treated with first-line paclitaxel and bevacizumab, Pizzuti et al. found that BMI did not affect overall response rate or disease control rate in the overall patient cohort, but higher disease control rate associated with BMI ≥ 25 kg/m2 in TNBC patients (147). Furthermore, in the NeoALTTO study treated with neoadjuvant lapatinib, trastuzumab, or their combination plus paclitaxel, obesity was associated with reduced access to pCR in HER2+ luminal BC patients, but not in HER2- cases (118). In a multicentre observational cohort study, Eriseld et al. suggested that class I obesity was correlated with a worse OS in HER2+ metastatic BC patients treated with pertuzumab and/or trastuzumab emtansine (148).

On the other hand, immunotherapy side effects in these patients caused by obesity should be closely monitored and effectively managed. In addition, in a retrospective cohort study, obesity might be a risk factor of cardiotoxicity in HER2+ BC patients receiving trastuzumab, indicating the necessity to prevent cardiotoxicity when receiving this regimen for patients with obesity and other risk factors (149). Furthermore, Wang et al. also addressed the predictive value of obesity and affirmed that obesity was an independent risk factor of trastuzumab-related cardiac toxicity in elderly BC patients who received trastuzumab therapy (150).

Totally, obesity may exert different effects depending on the tumor type, immunotherapy method, and patient cohorts, which is attributed to a variety of lifestyle, clinical and pathological factors, emphasizing the significance of obesity as an important evaluation factor for BC immunotherapy efficacy and side effects. These findings pave the way to future research in taking into account patient BMI, in clinical modification of combined immunotherapy strategies aimed at obtaining an ideal clinical outcome.

### Weight Management

Observational evidence has linked physical inactivity and obesity to an increased risk of BC and a poor outcome in BC. Recent studies have revealed that weight management or interventions *via* diet, physical activity, bariatric surgery could be important determinants of BC risk and outcomes (151–157). As Lee et al. reported that a 16-week of supervised aerobic and resistance exercise interventions could decrease the 10-year risk of cardiovascular disease in early BC women who were overweight or obese (158). In the study of Sweeney et al., a 16-week exercise significantly promoted the shoulder function in overweight or obese women after BC treatment (159).

Physical activity can improve the health outcomes of BC survivors by affecting insulin, IGF, insulin resistance, glucose metabolism, sex hormones, adipokines, inflammatory factors, oxidative stress, and DNA damage repair ability (160). A randomized controlled trial displayed that a 16-week aerobic and resistance exercise intervention could reduce M1 ATMs and adipose tissue secretion of the pro-inflammatory cytokines IL-6 and TNF-α, and increase M2 ATMs and secretion of anti-inflammatory cytokines such as adiponectin in obese post-menopausal BC survivors (161). The HFD-fed and ovariectomized mice showed a reduced anti-oxidative response and inflammation in TME, accompanied by the change of adiponectin and leptin in different tissues (162). It indicated that in the case of obesity, spontaneous physical activity could repress tumor progress by the interplay of adipose tissue, muscle and tumor tissue. Besides, diet and exercise interventions implemented in overweight/obese BC survivors may improve metabolic risk, insulin resistance and leptin biomarkers. Travier et al. designed a study that provided a 12-week diet and exercise program for overweight/obese BC survivors (154). the metabolic risk markers and insulin resistance indicators of BC survivors were significantly improved, while the reduction in leptin was not significant while adiponectin was significantly reduced. In another 18-month weight-loss trial of overweight/obese BC survivors women who lost >10% exhibited a significantly increased expression in serum adiponectin, as well as the improved adiponectin at 6 to 18 months despite weight regain (163). Achieving this level of weight loss appeared to be related to the regulation of adiponectin and was of great significance to the overall adipokine environment including the adiponectin/leptin ratio, insulin and PAI-1. Interestingly, Adams et al. enrolled 2 trials about the novel miRNAs that involved in BMI and weight loss in BC development (164). The results showed that obesity could affect the expression of tumor-associated miRNAs, highlight the potential mechanisms of the positive relationship between BMI and BC risk. Therefore, moderate-to-vigorous intensity resistance and aerobic exercise play positive roles in attenuating adipose tissue inflammation in obese BC patients, which could be beneficial for controlling BC.

## Conclusion

**Figure 2:**
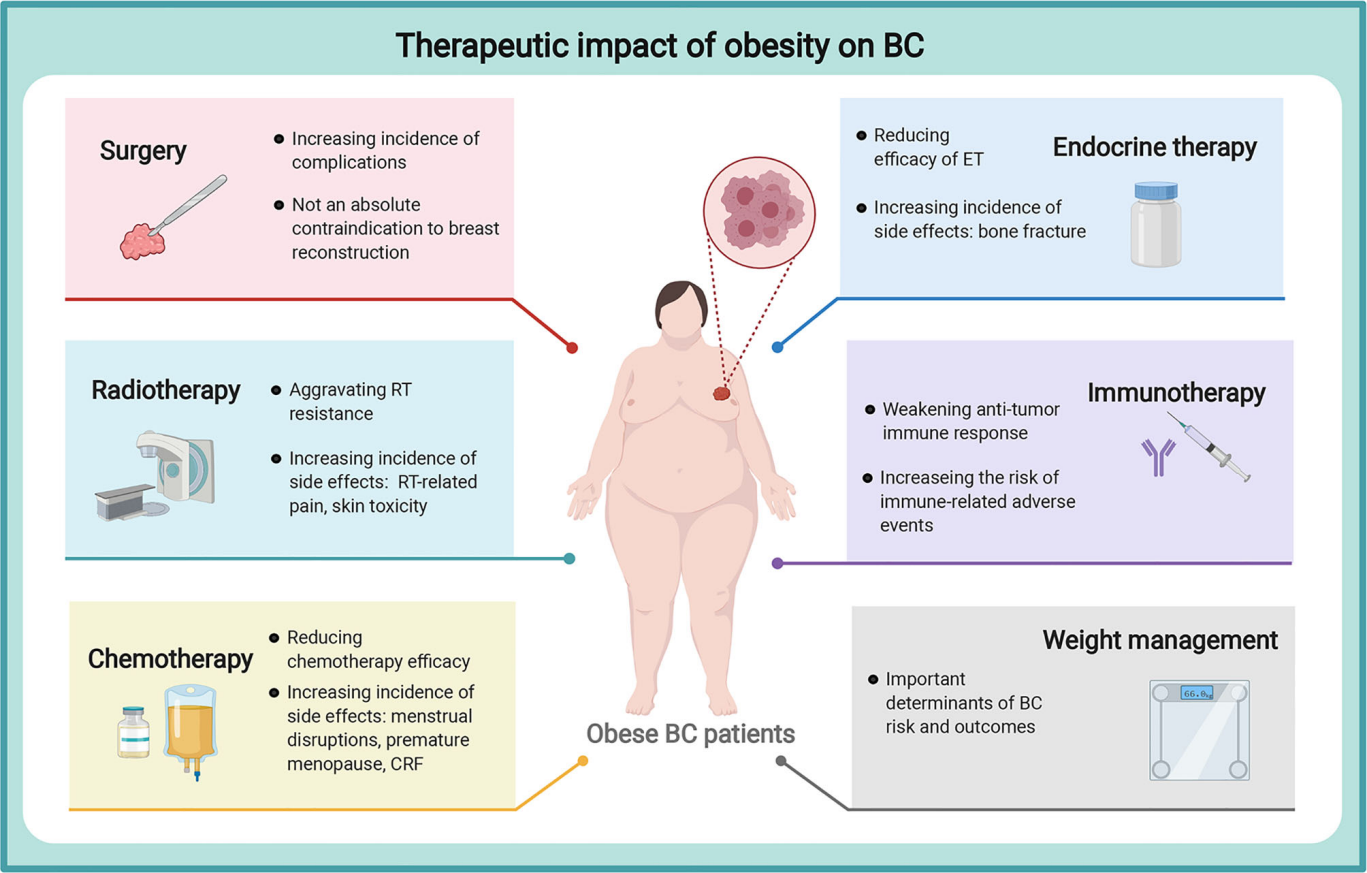
Therapeutic impact of obesity on breast cancer. Obesity could aggravate treatment resistance and potential drug side effects in various BC treatments, including surgery, RT, chemotherapy, ET, immunotherapy, and weight management, posing challenges for the maximum efficacy and minimum side effects of BC therapy. breast cancer, BC, breast cancer; RT, radiotherapy; CRF, cancer-related fatigue; ET, endocrine therapy. The figure was created with BioRender.com.

Collectively, a large increasing body of evidence strongly verifies obesity as a known risk factor for BC initiation and progression. The prevalence of obesity is posing challenges in BC incidence control and management considerations. Obesity is a very complex abnormal state, accompanied by various physiological and molecular alterations, which involves the complicated roles of adipokines (leptin, adiponectin, resistin, visfatin, and SFRP5), insulin, IGF, sex hormone, and chronic inflammation. These factors will synthetically affect cell proliferation, angiogenesis alteration, oncogene activation, oxidative stress, and immune cell dysfunction, which capable of regulating the behavior of BC cells and BC TME, ultimately. Targeting adipocyte-derived factors may be a feasible therapeutic approach to improving the prognosis of obese patients. On the other hand, obesity has recently been explored to present treatment resistance and potential drug side effects in various BC regimens, including surgery, RT, chemotherapy, ET, immunotherapy, and weight management, posing challenges for the maximum efficacy and minimum side effects of BC therapy (**Figure 2**).

Nevertheless, there are still some challenges to be addressed in terms of obesity and BC. First of all, although the interest in obesity and BC carcinogenesis gains momentum, the mechanism studies of obesity-triggered BC in favor of BC prevention and therapy are still investigational. Inflammation of adipose tissue may contribute to BC independently of obesity. More thorough and ongoing studies with large-scale in the future are warranted to decipher the interrelationships and roles that underlie the obesity-derived factors and BC conundrum. Secondly, in different BC subtypes, especially the high incidence TNBC and HER2+ BC, the relationship between obesity and prognosis is unclear and the conclusions are not quite consistent. This might attribute to the fact that obesity involves many confounding factors including age, the presence or absence of CLSs, population, menopausal status, and molecular type of the tumor. It means that the influence extent and difference of obesity on the treatment and prognosis of these BC subtypes remains to be clarified. Thirdly, obesity might counteract the benefits of BC treatment and raise the possible side effects. Some of the associations with adverse outcomes may have to do with poor therapeutic doses in obese patients due to dose-limiting toxicity. Attention needs to be paid to individualized dosing and close monitoring of treatment for BC patients with obese states. Of cause, lifestyle interventions *via* weight control, such as exercise, weight loss, and alimentary control, are considered as emerging approaches for better prevention and survival of BC patients. Fourthly, BMI is an index for weight in large amounts of studies. But BMI is defined only in relation to weight and height, which actually lacks body composition measure. Sometimes BMI might be inaccurate in defining obesity or emaciation. Therefore, the definition of body composition patterns and biomarkers related to the true nature induced by obesity is necessary for more accurate verification. Besides, key biomarkers of risk are still lacking in obesity-related BC prevention, although ongoing studies have been devoted to this area. The identification of more specific biomarkers will provide mechanistic insight into predicting the response to effect, adverse reaction, and resistance of combined targeted therapies of BC patients with obesity. At last, a comprehensive understanding of the biological mechanisms of obesity on treatment effectiveness and tolerance is necessary for maximizing the efficacy of BC therapy. Addressing the obesity state will allow the improvement of more personalized and effective prevention and treatment strategies for BC.

## Author Contributions

CZ, WH, and YX performed the literature search and wrote the manuscript. HW, QZ, and YPW conceived the project and revised the manuscript. DW, YCW, WL, MX, and YY edited the manuscript. All authors contributed to the article and approved the submitted version.

## Funding

This section acknowledges contributions from the China GuangHua Science and Technology Foundation (No. 2019JZXM001) and Wuhan Science and Technology Bureau (No. 2020020601012241).

## Conflict of Interest

The authors declare that the research was conducted in the absence of any commercial or financial relationships that could be construed as a potential conflict of interest.

## Abbreviations

BC, Breast Cancer; BMI, Body Mass Index; WHO, World Health Organization; TME, Tumor Microenvironment; IGF, Insulin-like Growth Factor; RT, Radiotherapy; ET, Endocrine Therapy; TNBC, Triple-negative BC; OS, Overall Survival; BMBC, Brain Metastases from BC; EMT, Epithelial-to-Mesenchymal Transition; PDX, Patient-derived Xenograft; obASCs, Obesity-altered Adipose Stem Cells; DIO, Diet-induced Obesity; CSC, Cancer Stem Cell; MDSCs, Myeloid-derived Suppressor Cells; FAO, Fatty Acid β-oxidation; HFD, High-fat Diet; SFRP5, Secreted Frizzled-related Protein 5; InsR, Insulin Receptor; RFS, Recurrence-free Survival; IGF-1, Insulin-like Growth Factor 1; ASCs, Adipocyte-derived Stem Cells; CCL2, CC Chemokine Ligand 2; COX-2, Cyclooxygenase-2; WAT, White Adipose Tissue; IL, Interleukin; TNF, Tumor Necrosis Factor; HIF-1α, Hypoxia-inducible Factor 1α; CLSs, Crown-like Structures; PGE2, Prostaglandin E2; miRs, micro-RNAs; CRP, C-reactive Protein; pCR, Pathological Complete Response; Dox, Doxorubicin; CRF, Cancer-related Fatigue; AKR, Aldo-keto Reductase; CBR, Carbonyl Reductases; NAC, Neoadjuvant Chemotherapy; ASCO, American Society of Clinical Oncology; AIs, Aromatase Inhibitors; Tx, Tamoxifen; CTGF, Connective Tissue Growth Factor; T-DM1, Trastuzumab Emtansine.
